# 785 Assessing The Kinematics Of Virtual Reality Gaming As Physical Therapy In Burn Patients

**DOI:** 10.1093/jbcr/irac012.336

**Published:** 2022-03-23

**Authors:** Cecil S Qiu, Scott Vocke, Joshua Rodriguez, Ryan Roemmich, Gregory Andre, Brooke Dean, Julie Caffrey

**Affiliations:** Johns Hopkins, Baltimore, Maryland; Johns Hopkins University, Baltimore, Maryland; Johns Hopkins Bayview, Timonium, Maryland; Johns Hopkins University, Baltimore, Maryland; Johns Hopkins Bayview Medical Center, Lutherville Timonium, Maryland; Johns Hopkins Bayview Medical Center, Baltimore, Maryland; Johns Hopkins, Baltimore, Maryland

## Abstract

**Introduction:**

Virtual reality (VR) gaming offers an immersive experience that can enrich physical therapy for the burn patient by introducing variability, enjoyment, and reward during functional motions of the upper extremities. In this study, we aim to support a proof-of-concept for VR gaming in upper extremity burn rehabilitation by characterizing shoulder and elbow kinematics during VR gaming in a healthy volunteer.

**Methods:**

A healthy volunteer without burn injuries played two games, a virtual rhythmic baton and virtual boxing game, on a commercially available VR gaming platform. Kinematics during play were assessed using two external cameras placed orthogonally, to the player’s front and left, so that 3-dimensional motion of the player’s left arm could be captured. Video of each gaming session was processed using an open-source perceptual computing software that dynamically tracks the user’s upper extremity during play. Kinematics at the left shoulder and elbow were characterized with respect to range of motion (ROM) and time spent in composite positions.

**Results:**

During the rhythmic baton game, the player achieved 157 degrees of elbow flexion ROM and 90 degrees shoulder elevation ROM. During the boxing game, the player achieved 156 degrees of elbow flexion ROM and 123 degrees shoulder elevation ROM. The baton game was associated with more time spent in the “rest” position (elbow extended with shoulder adducted, 60% of the game) while boxing was associated with more time in the “Guard” position (elbow flexed with shoulder elevated, 79% of the game) (Figure 1). Both games demonstrated simultaneous movement at both the shoulder and elbow during play.

**Conclusions:**

The two VR games investigated in this kinematic assessment challenged players to achieve a wide range of motion at the upper extremity with functional, multi-joint movements. These findings support a potential role for commercial VR gaming in burn rehabilitation and future research in burn patients is required to demonstrate its therapeutic value.

